# When Is Authoritarian Leadership Less Detrimental? The Role of Leader Capability

**DOI:** 10.3390/ijerph20010707

**Published:** 2022-12-30

**Authors:** Qiufeng Huang, Kaili Zhang, Yanqun Wang, Ali Ahmad Bodla, Duogang Zhu

**Affiliations:** 1 School of Political Science and Public Administration, Huaqiao University, Quanzhou 362021, China; 2 School of Business, East China University of Science and Technology, Shanghai 200237, China; 3 Nijmegen School of Management, Radboud University, 6525 AJ Nijmegen, The Netherlands; 4 School of Journalism and New Media, Xi’an Jiaotong University, Xi’an 710049, China

**Keywords:** authoritarian leadership, leader effectiveness evaluations, leader capability, task performance, affective organizational commitment

## Abstract

We developed and tested a moderated mediation model of the relationship between authoritarian leadership and employees’ task performance as well as their affective organizational commitment. Analyses of multilevel, multisource, and three-wave data from 99 supervisors and 341 subordinates showed that leader effectiveness evaluations mediated the time-lagged relationship of authoritarian leadership with employees’ task performance and affective organizational commitment. Moreover, when leader capability is high, it mitigates the negative relationship between AL and employees’ outcomes. Furthermore, the leader capability moderates the indirect relationship of authoritarian leadership with employees’ task performance and affective organizational commitment via leader effectiveness evaluation. This study contributes to leadership research and extends our understanding of how and under what circumstances AL is less detrimental to employees’ workplace outcomes.

## 1. Introduction

Authoritarian leadership (AL), defined as the extent to which leaders exert absolute authority and control over subordinates and demand unquestioning obedience, is a prevailing leadership style in current organizations [1,2,3]. Being considered to serve the function of ensuring employees’ compliance, AL was regarded to bring high performance [4,5]. Nevertheless, most empirical studies have found a negative relationship between AL and employees’ outcomes; AL has a destructive effect on employees’ intention to stay [6,7], work–family balance [8], employees’ creativity [9], extra-role performance [10,11,12], ethical behavior [13], work performance [14,15,16], and employees’ proactive behavior [10,17,18,19]. Together with the above, AL has been viewed as a kind of “dark side” leadership [13,20]. Considering AL prevalence in East Asian countries, it is interesting and vital to investigate how and when AL does not harm employees’ workplace outcomes or effectiveness [1,7,21]. 

To date, a dominant perspective to studying psychological mechanisms that link AL to employees’ outcomes and contextual factors that impact the effectiveness of AL focused on the attributes of subordinates. More explicitly, studies explained the mechanism of AL from an employee-centered perspective by showing how AL may influence employees’ cognitive and emotional evaluations, such as self-identity [22], perceived insider status [6], uncertainty evaluation [18], employees’ fear [9], work alienation [23,24], role perception [10], employee emotion suppression [8], and perceived powerlessness [19].Furthermore, a few studies focused on employees’ relational evaluations, such as trust in their supervisor [12,25,26] and LMX ambivalence [27]. In addition, with the employee-centered perspective, previous limited studies have shown that employees’ attributes, such as obedience tendency [28], individual role breadth self-efficacy [6], employees’ traditionality values [22], followers’ need for structure [21], employees’ power distance orientation [7], psychological capital [9], and employee moral efficacy [17], may work as essential boundary conditions influencing the outcomes of AL. 

Despite being insightful, an employee-centered perspective has the disadvantage of limiting the development of the system to understand the influence of AL. Employees might evaluate leaders when leaders show AL and how leaders’ own characteristics may potentially impact AL’s outcomes. Without the role of leaders in co-creating leadership, leadership is less likely to emerge [29]. Indeed, leaders and followers do not operate in isolation but mutually constitute each other: leaders react to and are enabled by their followers, whereas followers respond to and take direction from their leaders [7]. These concerns suggest that the outcome implications of AL cannot be captured by the employee-centered perspective. Therefore, it is vital to consider the nature of leaders in the context of understanding the impact of leadership [30]. Scholars such as Schaubroeck et al. call for more research on AL that goes beyond the role of the followers [6], emphasizing the necessity to bring an alternative perspective to understand the impact of AL on employee outcomes. Thus, our primary objectives are twofold: First, we move beyond the prior studies that mostly focused on employees’ feelings or cognitions [6,9,18,22,23,24]. This study shows how leader-related evaluations can work as the underlying mechanism in the relationship between AL and employees’ performance and attitudinal outcomes. Second, adopting a leader-centered perspective, our study shows that employees’ outcomes are contingent on the AL leaders’ characteristics or attributes. By doing so, we respond to the call on the contextual factors in affecting AL’s effectiveness [26,31]. 

Explicitly, based on a leader-centered perspective, we proposed that AL is negatively related to leader effectiveness evaluations, which may harm the employees’ task performance and affective organizational commitment. Moving beyond the leadership effectiveness criteria (e.g., leader task performance, leader relational performance, team performance) [32], we used employee subjective evaluation to show leader effectiveness. Such subjective evaluation can reflect employees’ overall judgment of the current leader’s effectiveness, which objective leadership effectiveness may not achieve. Considering the impact of employees’ identification with leaders [33,34], it is crucial to examine employees’ evaluation of leaders’ effectiveness in affecting their reactions to AL. 

Moreover, in Fiedler’s contingency theory [35], it is argued that autocratic leadership would be most effective in situations of either very low or very high control. A high-control situation would arise when the task structure is clear, a leader holds power, and leader–member exchange relations are high. The opposite is the case in situations of deficient control. Following the argument and considering a leader-centered perspective, the current study focuses on leaders’ power, which consists of leaders’ discretionary power to reward or punish, job-relevant expertise, and official status [36]. AL shares similarities with the reward and punishment component, and this study focuses on leaders’ job-relevant expertise, referred to as leader capability. Leader capability can offer a legitimate foundation for leaders’ official status. Thus, in a leading position, high capability offers individuals support to exercise power [37]. Therefore, when leader capability is high, it mitigates the negative relationship between AL and employees’ outcome. 

Our study contributes to the literature on AL in several aspects. First, by examining the leader-oriented process and condition, this study enriches the implication of AL leader-centric research. It explores when AL may not necessarily bring harmful results. Different from the previous studies focused on the follower-centered perspective [6,9,18,23,24], this study contributes to the discussion on the leader characteristics in exploring the impact of AL. Second, by showing the mechanism (i.e., leader effectiveness evaluation) in linking AL and employees’ workplace outcomes, this study offers a different view in examining the underlying mechanism of AL and employees’ outcomes. Third, by adopting the leader-centered perspective, we present empirical evidence of interactive effects between leadership characteristics (leader capability) and AL on employees’ outcomes, answering the question of under what circumstances AL is less detrimental to employees’ workplace outcomes.

## 2. Theory and Hypotheses

### 2.1. Authoritarian Leadership, Leader Effectiveness Evaluation, and Employee Outcomes

Authoritarian leadership refers to a leader asserting strong authority and control over subordinates and demanding unquestioned obedience. Leaders who are highly authoritarian apply strict discipline in supervising subordinates’ work assignments and highlight authority by making independent decisions [3,38]. As such, authoritarian leadership is usually viewed as a “controlling leadership style” that may be detrimental to employees’ performance [18,21,26]. For instance, studies have demonstrated that an authoritarian leader generally has low levels of power- and information-sharing behaviors [6]. Moreover, employees are more likely to be punished if they do not strictly follow leaders’ instructions [22].

Employees’ perceptions of leaders’ ability to fulfill responsibilities in a leadership position have led to leader effectiveness evaluation [33,39]. Such conceptualization differs from leader effectiveness criteria because it indicates leaders’ own task and contextual performance outcomes or the group performance [40,41]. For instance, taking group performance as the indicator of leader effectiveness, studies have found that when the group performs well and fulfills its goals, leaders are considered effective [42,43]. Differently, leader effectiveness evaluation focuses on employees’ subjective evaluation of the current leader’s effectiveness. When leaders are evaluated as highly effective, they are considered to endorse the leadership and fulfill their responsibilities [39,44]. Prior studies have shown that when leaders demonstrate consideration and care toward followers, such as self-sacrificing behaviors [45] or transformational leadership behavior [46], they are more likely to be evaluated as having high work effectiveness [32,39]. 

Following the above argumentation, we propose that AL is negatively related to leader effectiveness evaluations for several reasons. First, high authoritarianism is often viewed as the main power asymmetry between leaders and their followers [47] and is considered an abuse of power that derives from self-centered motives and a quest for personal benefits [21,22]. In this sense, leaders are less likely to gain followers’ trust [12,26], which composes the critical criteria in leader effectiveness evaluation [39]. Second, authoritarian leadership implies that leaders view their employees as incapable of performing without leaders’ directions [48,49]. As such, leaders often require their followers to obey their instructions without doubts and regularly monitor followers’ performance. Under such a situation, followers would be less likely to feel they are being given the opportunities and support for personal development, which also composes a key to leader effectiveness evaluation. Third, an authoritarian leader may put more emphasis on work outcomes while offering limited information and explanation for their decisions, which impedes employees’ understanding of supervisors’ work efficiency. Moreover, with limited dyadic interactions between leaders and followers [4,18], chances of misunderstandings between the dyads may be enhanced to ultimately harm employees’ favorable evaluations of their supervisors [46]. Thus:

**Hypothesis** **1.** *Authoritarian leadership is negatively related to leader effectiveness evaluation*.

Although studies have depicted that effective leaders can enhance employees’ willingness to perform a job-related task and to cooperate toward collective goals [45,50], empirical research is still limited. Hence, this study further examines the outcomes of the leader effectiveness evaluations. When leaders are considered effective, employees are likely to have high task performance. When leaders are perceived as highly effective, employees may show strong identification with leaders [44]. With a strong identification with their leaders, employees are more likely to take cooperative actions that can also benefit their leaders [34,51]. In addition, employees may also want to be liked, noticed, and valued by leaders who are considered effective; therefore, they may exert themselves to a high-level task performance to gain leaders’ attention. Taking the above arguments together:

**Hypothesis** **2a.** *Leader effectiveness evaluation is positively related to followers’ task performance*.

When leaders are considered highly effective, employees may also show high affective organizational commitment. Affective organizational commitment refers to an “emotional attachment to, identification with, and involvement in the organization” [52] and is seen as employees’ positive attitude toward organizations. When leaders are evaluated as being of high effectiveness, employees generate high identification with the leader. This spills into an emotional bond with the leader to the organization, as leaders usually work as the agents of the organization [53,54]. Furthermore, employees supervised by effective leaders would work more fluently without annoying disturbances or distractions because of the high involvement of aspiring to contribute to the organization. Taking the arguments together, we hypothesize:

**Hypothesis** **2b.** *Leader effectiveness evaluation is positively related to followers’ affective organizational commitment*.

Based on Hypothesis 1 and Hypothesis 2 above, we thus come to the mediation hypotheses:

**Hypothesis** **3a.** *Leader effectiveness evaluation mediates the negative relationship between authoritarian leadership and followers’ task performance*.

**Hypothesis** **3b.** *Leader effectiveness evaluation mediates the negative relationship between authoritarian leadership and followers’ affective organizational commitment*.

### 2.2. The Moderating Role of Leader Capability

The contingency leadership theory has been widely applied to explain the effectiveness of certain leadership styles. It predicts that a leader’s effectiveness lies in a “match” situation. Specifically, a leader’s effectiveness is based on two main factors: a leader’s attributes, referred to as task or relationship-oriented style, and a leader’s situational control, referred to as task structure, leader–follower relations, and position power. Task-oriented leadership will be more successful either in high- or low-control situations, while relationship-oriented leadership will be more successful in the moderate-control condition [55]. Following the above, AL would be viewed as effective in situations of very high control, shown by a clear task structure, high leader power, or high leader–member relations. In line with our leader-centered perspective, we focused on leaders’ power.

Leaders’ power reflects their discretionary power to reward and punish, job-relevant expertise, and official status [56]. Since AL also shows leaders’ discretionary power to reward and punish (e.g., high AL can exert more substantial authority and require subordinates’ unquestioned obedience), we focused on leaders’ job-relevant expertise. From a power perspective of leadership [57], leaders are considered to hold high official status when they have high job-relevant expertise. Therefore, this study examines leader job-relevant expertise and capability to examine AL effectiveness evaluation. Leader capability shows the ability of the leader to perform functions necessary to accomplish specific leadership roles and responsibilities [58,59]. Leader capability is not a fixed act or simply knowing what to do; instead, it involves the generative capability to organize different skills toward changing tasks. Therefore, capability is viewed as individuals’ knowledge, expertise, and general wisdom needed to fulfill their responsibilities in the workplace. 

Based on the above reasoning, we expect that when leaders have high capability, the negative relationship between AL and leader effectiveness evaluation is mitigated. Following the contingency theory, when leaders that hold high power indicate high capability, AL is regarded as effective. On the one hand, a capable person is more likely to be viewed as legitimate to becoming the leader, which makes his/her power impactful and effective [60], mitigating the negative effect of AL. Thus, a leader with high capability tends to make centralized decisions due to his/her high professional experience and knowledge; thus, a controlling leadership style can be viewed as acceptable and legitimate [61]. In addition, research showed that when leaders are of high capability, a controlling leadership style may be viewed as less distractive if not attractive [45]. Drawing on the contingency leadership theory, which proposed that leaders are more likely to achieve effectiveness with high power, we hypothesize that the leader capability can only mitigate the negative impact of AL rather than reverse the negative impact. Thus, the contingency leadership theory proposes that a high control condition contains several factors besides leader power; hence, we consider that even with high leader power, it may not be strong enough to reverse the negative impact of AL. In addition, following the AL literature, most empirical studies have found that contingent factors can only mitigate the negative impact of AL, while less likely to reverse the negative relationship [6,22]. Taken together, we hypothesize:

**Hypothesis** **4.** *Leader capability moderates the negative relationship between authoritarian leadership and leader effectiveness evaluation, such that the negative relationship is mitigated when leader capability is high*.

Thus far, we have developed theoretical underpinnings for the mediating effect of leader effectiveness evaluation and for the contingent effects of leader capability. Authoritarian leadership is indirectly related to task performance and affective organizational commitment via the role of leader effectiveness evaluations (H3a and H3b). Leader capability moderates the negative relationship between authoritarian leadership and leader effectiveness evaluations (H4). Integrating the rationales for the hypotheses suggests a moderated mediation model. Therefore, the indirect influence of authoritarian leadership on task performance and affective organizational commitment through leader effectiveness evaluation will be weakened when leaders hold high capability compared to when it is low. This leads to the following hypotheses:

**Hypothesis** **5a.** *Leader capability moderates the indirect effect of authoritarian leadership on followers’ task performance via leader effectiveness evaluation, such that this negative indirect effect is weakened under high leader capability but is enhanced under low leader capability*.

**Hypothesis** **5b.** *Leader capability moderates the indirect effect of authoritarian leadership on followers’ affective organizational commitment via leader effectiveness evaluation, such that this negative indirect effect is weakened under high leader capability but is enhanced under low leader capability*.

**Figure 1:**
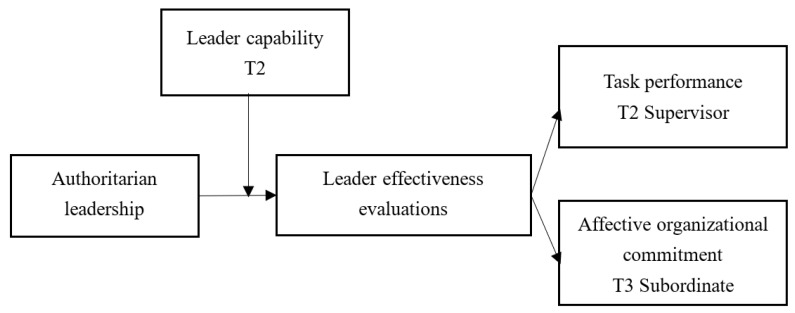
The theoretical model.

In sum, previous studies holding a negative attitude toward AL styles stems from the fact that AL has predominantly adverse effects on followers [6,7,8,9,10,11,12,13,14,15,16,17,18,19]. However, recently, empirical evidence that AL may not necessarily bring harmful results started to accumulate with exploring the boundary conditions and mechanisms in influencing the outcomes of AL by the follower-centric perspective [6,9,18,22,23,24]. However, leadership arises from a number of factors, such as the personal qualities of both leaders and followers, to impact how followers react to leadership [30]. In addition, research needs to pay more attention to the leaders’ characteristics to identify the effects of AL on followers’ outcomes; findings cannot be explained by a mere follower-centric perspective. Thus, building on a leader-centric perspective, we developed our hypotheses and posited that AL is less detrimental to employees’ task performance and affective organizational commitment when leader capability is high, and leader effectiveness evaluation works as a mediating variable in the link between AL and followers’ outcomes. The theoretical model is shown in Figure 1 below.

### 2.3. Methods Review

To test our model, we followed prior studies by applying the positivism paradigms. Positivism paradigms assume that individuals’ behavior is intrinsically connected, and individuals are very clear about their own motivations and meanings. Using rigorous methods (e.g., conceptualization, measurement), the researcher could explore and understand the insider’s interpretation of behavior positivism paradigms. This highlights research for causes and explanations that support the causality explanation, emphasizing the generality and universality of the theory. Moreover, positivism paradigms advocate that the empirical methods of the natural sciences (such as observation, experiments, questionnaires surveys, and comparisons) should be used to study social phenomena [62]. In the current study, we adopt the questionnaire surveys to test our theoretical model by following the positivism paradigm. 

In addition, we use a multilevel analysis method to collect the data. The multilevel analysis method can help us to rule out the team-level impact, as one leader needs to supervise several employees, and leaders’ evaluation of employees’ performance may be biased based on leaders’ characters. For instance, team A’s leader may have a general tendency to evaluate employees in a more positive manner, while team B’s leader may have a general tendency to evaluate employees in a negative manner. Thus, we applied the multilevel approach to show how employees’ performance outcomes are based on their own work experience rather than the impact of leader’s character. Such methodology has also been widely adopted in multilevel studies when the aim is to rule out the nonindependence effect [18,63,64].

## 3. Methods 

### 3.1. Sample and Procedure

Data were collected from an architectural design company (whose principal business was designing administration buildings, parks, and public lounge areas) located in an eastern region of China. The human resource department assisted with introducing the study requirement and soliciting voluntary participation from employees and their direct supervisors in working teams. We guaranteed the confidentiality of all the responses.

At Time 1, employees reported their demographic information, power distance orientation, and authoritarian leadership. At Time 2 (six weeks later), employees evaluated leaders’ capability, leaders’ integrity, and leaders’ effectiveness. Moreover, at Time 2, their direct supervisors were required to evaluate employees’ task performance. At Time 3 (six weeks later), employees evaluated their affective commitment toward the organization. Of the respondents, 413 employees from 110 teams and their team leaders agreed to participate in the study initially. At Time 1, 388 employees from 103 teams completed the first survey, with a response rate of 93.95%. At Time 2, we distributed surveys to those who finished the Time 1 survey and their team leaders. We received matched surveys from 341 employees and 99 leaders (response rate: 87.89%). At Time 3, we further distributed surveys to employees who had finished the Time 1 and Time 2 surveys. This provided us with responses from 341 employees. Each team leader evaluated 2 to 6 followers. Of the participants, 72.73% were male; the average age was 36.58 years (SD = 9.89), the average education was 13.84 years (SD = 2.82), and the average dyadic tenure with the supervisor was 3.69 years (SD = 2.96).

### 3.2. Measures

We followed Brislin’s translation-back procedure to translate the scales from English to Chinese [65]. Unless otherwise specified, all items were rated with a seven-point Likert scale ranging from 1 (strongly disagree) to 7 (strongly agree).

Authoritarian leadership. Authoritarian leadership was measured using a nine-item scale from Chen and Farh [66]. Employees were asked to rate the leadership style of direct supervisors. A sample item was “My immediate supervisor has asked me to obey his/her instructions completely.” The Cronbach’s alpha for this scale was 0.89. 

Leader capability. Four-item scale was adopted from Evans and colleagues’ [58] study. The items for this scale were “My supervisor is very intelligent”, “My supervisor is very competitive”, “My supervisor is very competent”, and “My supervisor is very confident”. The Cronbach’s alpha for this scale was 0.91.

Leader effectiveness evaluation. A five-item scale was adopted from van Knippenberg and van Knippenberg [39] and Cicero and colleagues [67]. Items for this scale were “My supervisor is an excellent leader”, “I put my trust in my supervisor”, “My supervisor is very effective as a leader”, “My supervisor influences my level of commitment effectively”, and “Overall, I feel a good level of agreement with my supervisor”. The Cronbach’s alpha for this scale was 0.96. 

Task performance. A four-item scale was adopted from Methot, LePine, Podsakoff, and Christian [68]. A sample item for this scale was “This employee fulfills the responsibilities specified in his/her job description.” The Cronbach’s alpha for this scale was 0.90.

Affective organizational commitment. A six-item scale was adopted from Allen and Meyer [69] to measure affective organizational commitment. A sample item for this scale was “I would be happy to spend the rest of my career with this organization.” The Cronbach’s alpha for this scale was 0.94.

Control variables. Employees’ age, gender, education, and dyadic tenure with the supervisor were controlled since these variables have been considered to impact employees’ identification with the supervisor [32]. The measures of these demographic variables were obtained from the subordinates. Gender was coded as a dummy variable (1 = male, 0 = female). Education was measured by the actual years of school study. Age and dyadic tenure were both measured with objective data. In addition, employees’ general acceptance of authority should be controlled. In the current study, we controlled for employees’ power distance orientation. Power distance orientation was adapted from Farh, Hackett, and Liang’s six-item scale [70]. A sample item was “Supervisors should seldom ask for opinions of followers”. The alpha coefficient was 0.92. In addition, to rule out the impact of leader integrity, we also controlled for leader integrity evaluation. Leader integrity evaluation was measured with six-items from Mayer and Davis [71]. A sample item is “My supervisor has a strong sense of justice.” The alpha coefficient was 0.94.

### 3.3. Analytical Strategy

We tested all the hypotheses using Mplus 7.2 [72]. Because each participant’s data were nested within a supervisory unit together with other participants, we thus followed the prior studies to apply the multilevel analysis [18,63,64]. In particular, we employed the “Cluster” and “Type = Twolevel” syntax to account for nonindependence. With this approach, the standard errors are adjusted to account for nonindependence due to individuals’ clustering within a supervisory unit. To obtain accurate indirect effects, we tested these effects using the Monte Carlo resampling method [73]. Using information from the asymptotic covariance matrix of estimated model coefficients, this method repeatedly simulates indirect effects to obtain a distribution of the indirect effect.

The interaction terms were calculated based on the group-mean-centered variables [74]. For the moderated mediation hypotheses, we also used Monte Carlo simulation and constructed 95% confidence intervals (CIs) to test the indirect effects at high (1 SD above mean) and low (1 SD below mean) levels of the moderator [73]. We utilized 20,000 re-samplings for each confidence interval. The effect is significant (*p* < 0.05) if the confidence intervals exclude zero.

## 4. Results

### 4.1. Preliminary Analysis

Given the nested structure of our data, we used Mplus 7.2 and conducted a set of two-level confirmatory factor analyses (CFA) to examine the discriminant validity of the variables in our hypothesized model and power distance orientation. We specified parameters for the within-level model and kept the between-level empty. The measurement model demonstrated an adequate fit for the data. The results showed that the seven-factor model (with two control variables) fit the data quite well: χ^2^ (719) = 1040.06, *p* < 0.01, root-mean-square error of approximation (RMSEA) = 0.04, comparative fit index (CFI) = 0.97, standardized root-mean-square residual (SRMR) (within) = 0.05. Items all significantly loaded on their respective latent factors (standardized factor loadings ranging from 0.59–0.92, *p* < 0.01). The correlations among factors range from −0.33 to 0.67. The hypothesized measurement model exhibited a better fit than alternative models in which we loaded items from different measures onto one latent factor (∆χ^2^ ≥ 665.46, *p* < 0.01). These results offer support for the discriminant validity of the studied variables.

Before testing the hypotheses, we examined the levels of within- and between-group variance for leader endorsement and employee performance outcomes. One-way random-factor analysis of variance results showed that the between-group variance was significant for leader effectiveness (F (98, 242) = 4.45, *p* < 0.01); task performance (F (98, 242 = 4.75, *p* < 0.01); affective organizational commitment (F (98, 242) = 3.03, *p* < 0.01). We further computed intra-class correlation indexes (ICCs). Results indicated that there was a substantially high portion of between-group variance for leader effectiveness (ICC (1) = 0.50); for leader-rated task performance (ICC (1) = 0.52); and for affective organizational commitment (ICC (1) = 0.37). The ICC results indicate significant nonindependence in our data. Therefore, a multilevel model is needed.

Table 1 presents the zero-order correlations and descriptive statistics for the studied variables. As shown in Table 1, authoritarian leadership has negative relationships with leader effectiveness (r = −0.20, *p* < 0.01), employees’ task performance (r = −0.26, *p* < 0.01), and affective organizational commitment (r = −0.13, *p* < 0.05). Leader effectiveness has positive relationships with employees’ task performance (r = 0.53, *p* < 0.01) and affective organizational commitment (r = 0.64, *p* < 0.01). These findings offer preliminary support for our hypothesized relationships.

### 4.2. Hypotheses Testing

The unstandardized results of the hypothesized model are presented in Table 2. As shown in the results, authoritarian leadership was negatively related to leader effectiveness (γ = −0.15, SE = 0.08, *p* < 0.05), yielding support for Hypothesis 1. Leadership effectiveness evaluation was positively related to employees’ task performance (γ = 0.36, SE = 0.08, *p* < 0.05), and leadership effectiveness evaluation was also positively related to employees’ affective organizational commitment (γ = 0.58, SE = 0.11, *p* < 0.05), yielding support for Hypothesis 2a and Hypothesis 2b.

Hypothesis 3a and 3b suggest the mediation effect between authoritarian leadership and employees’ task performance and affective commitment via the role of leadership effectiveness evaluation. To estimate the indirect effects, we used 20,000 Monte Carlo replications [75]. Results showed significant negative indirect effects between authoritarian leadership and employees’ task performance via leader effectiveness (estimate = −0.06, 95% CI = (−0.1353, −0.0008)) and between authoritarian leadership and employees’ affective organizational commitment via leader effectiveness (estimate = −0.09, 95% CI = (−0.1828, −0.0007)). Therefore, Hypotheses 3a and 3b were supported.

**Figure 2:**
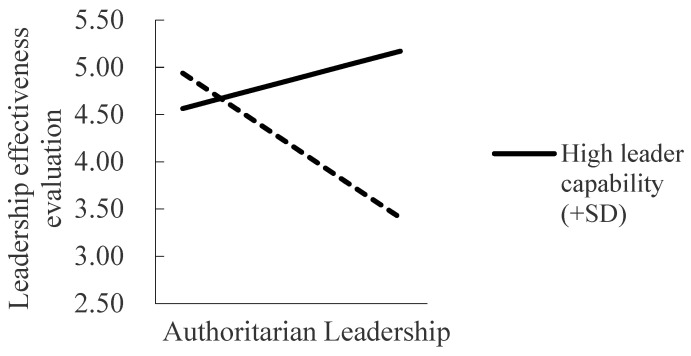
The relationship between authoritarian leadership and leader effectiveness under the conditions of low and high leader capability.

Hypothesis 4 suggests the moderating role of leader capability in the relationship between authoritarian leadership and leader effectiveness. The interaction between authoritarian leadership and leader capability was significant in predicting leader effectiveness (γ = 0.31, SE = 0.12, *p* < 0.05). We present the interactive pattern in Figure 2. Simple slope tests showed that authoritarian leadership was negatively related to leader effectiveness evaluation when leader capability was low (simple slope = −0.50, SE = 0.18, *p* < 0.01), but it was not significant when leader capability was high (simple slope = 0.19, SE = 0.13, *p* > 0.05). The difference between these simple slopes was 0.69 (SE = 0.27, *p* < 0.05). Thus, Hypothesis 4 was supported.

We then used the Monte Carlo method to test the moderated mediation effects hypothesized in Hypotheses 5a and 5b. Results showed that the indirect effect between authoritarian leadership and task performance via leader effectiveness was negative when leader capability was low (estimate = −0.18, 95% CI = (−0.3791, −0.0388)), but it was not significant when leader capability was high (estimate = 0.07, 95% CI = (−0.0225, 0.1790)). The difference between high and low conditional indirect effects was significant (difference = 0.25, 95% CI = (0.0451, 0.5325)). Thus, Hypothesis 5a was supported. The indirect effect between authoritarian leadership and affective organizational commitment via leader effectiveness was negative when leader capability was low (estimate = −0.29, 95% CI = (−0.4754, −0.0978)), but it was not significant when leader capability was high (estimate = 0.11, 95% CI = (−0.0401, 0.2441)). The difference between these conditional indirect effects was significant (difference = 0.40, 95% CI = (0.1094, 0.6698)). Thus, Hypothesis 5b was supported.

## 5. Discussion

### 5.1. Result Discussions

Taking a leader-centered perspective, this study examined when AL is less detrimental to employees’ task performance and affective organizational commitment, incorporating leader effectiveness evaluation as a mediating variable and leader capability as a moderating variable. A multilevel, multisource, and three-wave study was conducted. Based on the data from 99 supervisors and 341 subordinates, this study tested the hypotheses and revealed several essential conclusions that may contribute to the existing literature, as follows.

First, authoritarian leadership was negatively related to leader effectiveness, which in turn harmed employees’ task performance and affective organizational commitment. The results are consistent with the previous finding that AL was negatively related to followers’ outcomes [12,48], such as work–family balance [8], employees’ creativity [9], extra-role performance [10,11,12], ethical behavior [13], work performance [14,15,16], and employees’ proactive behavior [10,17,18,19]. Our study has found that AL adversely influences leader effectiveness evaluation, employees’ task performance, and affective organizational commitment, and we have empirically supported this finding.

Second, leader effectiveness evaluation was positively related to employees’ task performance and affective organizational commitment. Earlier studies on leader effectiveness evaluations have mainly focused on its antecedents while ignoring its outcomes [32,39,76]. Therefore, empirical evidence is still limited in demonstrating leader effectiveness that can foster positive outcomes. Hence, by empirically examining how leader effectiveness evaluation may be associated with employees’ task performance and affective organizational commitment, our study offers empirical evidence of the positive effect of perceived leader effectiveness.

Third, leader effectiveness evaluation mediates the negative relationship of AL with followers’ task performance and affective organizational commitment. This is different from prior studies, which adopted an employee-centered perspective and showed the mediating effect of self-identity [22], perceived insider status [6], uncertainty evaluation [18], employees’ fear [9], work alienation [23,24], role perception [10], employee emotion suppression [8], perceived powerlessness [19]; or their relational evaluations, such as trust in their supervisor [12,25,26] and LMX ambivalence [27] in linking AL and employees outcomes. Our study has recognized the vital role of leader effectiveness evaluation as the mechanism linking AL and employees’ workplace outcomes, explaining why AL harms subordinates’ task performance and affective organizational commitment from the leader-centered perspective.

Fourth, leader capability moderates the effect of AL on followers’ leader effectiveness evaluation, as well as moderates the indirect effect of AL on followers’ task performance and affective organizational commitment via leader effectiveness evaluation. More explicitly, when leaders’ capability is perceived as high, the negative relationship between AL and leader effectiveness evaluation is insignificant. Moreover, the indirect relationships between AL and employees’ task performance and affective organizational commitment were insignificant when leaders’ capability was high. The result is also consistent with previous studies showing that contextual factors can make AL less detrimental to employees’ workplace outcomes. For instance, Shen and colleagues [22] found that when a group believes in traditional values, the negative relationship between AL and employees’ relational identity is usually mitigated. Koveshnikov and colleagues [7] found that in the high-power distance context of Russia, AL reduces turnover intentions only among followers with a highly perceived prototype of a leader. Harms and colleagues [21] summarized followers’ characteristics (e.g., need for structure) and situational factors (e.g., resource availability) as important boundary conditions to impact the outcomes of AL.

### 5.2. Conclusions

The prevalence of AL in organizations has gained increasing attention in research. Nevertheless, most efforts have been put into an employee-centered perspective to show when and why AL may not harm employees’ workplace outcomes. Our study adopts a leader-centered perspective and showed that AL can harm employees’ task performance and affective commitment via the role of leader effectiveness evaluation; however, the above negative relationship is contingent on leaders’ capability. In other words, when leaders are considered as being of high capability, they are more likely to be legitimized for engaging in authoritarian behaviors.

### 5.3. Limitations and Further Research

Our study has a few limitations that provide suggestions for future research directions. First, using contingency leadership theory, our study only focused on the role of leader power, probed by leader capability, as the contingent factor to influence the effectiveness evaluations toward AL. Yet, it may also be possible that other factors may impact the effect of AL, such as leader integrity, and may work as an important conditional factor to influence employees’ evaluations toward AL [71]. In the current study, we performed a supplementary analysis to further show the moderating role of leader integrity. Results showed that the interaction between AL and leader integrity on leader effectiveness evaluation was marginally significant (B = 0.21, SE = 0.13, *p* > 0.10), which indicates that leader capability seems to account for more credits than integrity. However, we suggest that future studies integrate the two components into different contexts to examine the relationship between AL and employees’ outcomes. Moreover, as suggested in contingency leadership theory, task structure and leader–member relations may also work as important contingent factors to impact the effectiveness of AL. We suggest that future studies incorporate these two factors to examine the effective of AL.

Second, we found that under high leader capability, AL can be less detrimental. Yet, we did not find that high leader capability can reverse the negative impact of AL to achieve effectiveness (i.e., under high leader capability, the relationship between AL and leader effectiveness evaluation is 0.21, SE = 0.13, *p* > 0.05). In other words, the current study only found that, under certain conditions, the negative impacts of AL on leader effectiveness evaluation as well as employees’ workplace outcomes are attenuated but are not reversed to positive ones. Such findings may offer insights for future research. On the one hand, future studies may explore different underlying mechanisms or contingent factors in linking AL and employees’ outcomes to show when AL can achieve positive outcomes. For instance, Schaubroeck and colleagues [6] hypothesized that AL could be positively related to employees’ insider status evaluations under high-team-power distance values. Similarly, Huang, Xu, Chiu, Lam, and Farh [77] found AL can be effective during economic crisis. On the other hand, according to contingency leadership theory, the interplay of an explicit task structure, high leader–follower relations, and together with a leader’s high power (not only shown by high capability but high integrity) may be more powerful to reverse the negative impact of AL. Hence, future research can incorporate these different factors to show when AL may be less detrimental or to achieve effectiveness.

Third, from a leadership-centric perspective, this study focused on the leaders’ characteristics to explore how and when AL does not harm employees’ workplace outcomes or effectiveness; however, the interactive effect of leaders’ personal qualities and followers’ characteristics on followers’ workplace outcomes from both leader- and follower-centric perspectives should also be further explored. In fact, leaders and followers do not operate in isolation but mutually constitute each other [7]. Scholars such as Lord et al. [30] also argued that interactional factors such as the personal qualities of both leaders and followers would impact followers’ reaction to leadership [30]. Our knowledge about the empirical evidence of the interactive effects of leaders’ personal qualities and followers’ characteristics on the effects of AL leadership remains limited.

Fourth, our study is conducted in the Chinese context, where cultural values such as collectivism and power distance may affect how employees react to authoritarian treatment. For instance, studies on AL have suggested that cultural issues might be significant since different societies can tolerate authority differently, and AL may also have different popularity in different cultures. As Chinese culture generally expects power inequality [1], our findings may not be generalized to all situations. We suggest future studies conduct investigations in other cultural contexts to show the generalizability of the current findings and whether leaders’ authoritarian behaviors may enact different reactions from different cultures.

Fifth, we proposed power asymmetry, perceived control, and leaders’ information sharing in explaining how AL influences leaders’ effectiveness evaluation but did not test those mechanisms. Thus, future research could empirically explore whether they mediate the relationship between AL and leaders’ effectiveness evaluation.

Finally, methodologically, all hypotheses were tested using the questionnaire method, and the lack of three time-lagged or experimental research designs calls for caution regarding the accurate conclusion about the causal relationships between main variables. Future research should seek to refine and strengthen the methodology, for example, by collecting data on the variables at three time points or adopting the experimental research design, to better understand the proposed causal relationships between variables.

### 5.4. Implications

This study contributes to the literature in several ways. First, by examining the leader-oriented process and conditions, this study contributes to the AL literature by further demonstrating when AL may not necessarily bring harmful results. Initially acknowledged to foster employee compliance, AL was considered to foster high-performance outcomes [1,4]. However, empirical studies have illustrated that AL has a detrimental effect on followers [6,7,8,10,11,12,14,15,16,17,18,19] or even team outcomes [31]. However, some recent studies suggested that AL might not lead to adverse outcomes, or in some cases it might even lead to positive relationships between AL and followers’ outcomes [25,78,79]. This variability in research conclusions has promoted the call to further identify different underlining mechanisms in linking AL and employee outcomes and conditional factors that may affect the impact of AL [6]. Therefore, it becomes theoretically appealing to explore further when and why AL may not lead to adverse employee reactions, especially considering the prevalence of AL in current organizations. The emerging literature that takes a follower-centric perspective has suggested that the nature of followers would impact the effect of AL [6,9,18,22,23,24]. Overall, this study enters the dialogue with previous research on the questions about when and how AL does not harm employees’ workplace outcomes. Distinct from prior follower-centric research, our study takes the leader-centric approach to examine the effect of leader-oriented processes and conditions in linking AL and employee outcomes. More generally, our study has critical implications for AL leader-centric research and suggests the importance of considering the characteristics of a leader that may explain when and how AL does not harm employees’ workplace outcomes or effectiveness.

Second, our leader-centered perspective also enriches the potential mechanisms for linking AL and employees’ workplace outcomes and opening the “black-box” of the underlying processes from AL to employees’ outcomes, complementing previous findings. Prior research has generally adopted an employee-centered perspective to study the underlying mechanism and using self-identity [22], perceived insider status [6], uncertainty evaluation [18], employees’ fear [9], work alienation [23,24], role perception [10], employee emotion suppression [8], perceived powerlessness [19], trust in their supervisor [12,25,26], and LMX ambivalence [27] as the mechanisms to explain the impact of AL on employees’ performance outcomes [6,14,22]. For instance, under the social exchange lens, studies have found that AL would harm trust in supervisors, which then hampers employees’ in-role and extra-role performance [11,12]. Focusing on employees’ self-evaluation, studies have also found that AL would harm employees’ self-concept (e.g., organizational-based self-esteem), which then harms their performance [14,15,16]. Recent studies have also found that AL triggers employees’ uncertainty evaluation toward the context, which then inhibits employees’ proactive behavior [10,17,18]. Despite insightful findings, it was overlooked how AL may influence leadership effective evaluation, which has been critical to employees’ workplace outcomes [34,44]. Hence, by the leader-centered process, this study shows that leader effectiveness evaluation is an important mediating mechanism for the relationship between AL and employees’ outcomes, which offers a complementing view of AL’s impact on employees’ outcomes.

Third, with the consideration of leader capability as a moderator, this article also contributes to the research on leader characteristic moderators of the relationship between AL and employees’ outcome by following a leader-centered perspective. In particular, a number of studies have examined the critical contingencies that can affect the functions of AL, such as obedience tendency [28], individual role breadth self-efficacy [6], employees’ traditionality values [22], followers’ need for structure [21], employees’ power distance orientation [7], psychological capital [9], and employee moral efficacy [63], and found that they may influence the outcomes of AL [6,15,28]. However, most of them considered employees’ differences from an employee-centered perspective, which may restrict our understanding of the role of power holders and leaders’ characteristics in impacting their power effectiveness. Although a few studies highlighted how different leadership styles might compensate for a controlling leadership style (e.g., benevolent leadership) [14,80], it remained primarily unknown how relatively stable leader characteristics may influence leader authoritarianism. Therefore, focusing on relatively stable leader characteristics of leader capability in the relationship between AL and its outcomes, this study addressed the issue and enriched our understanding of how leaders’ capability influenced AL effectiveness. More explicitly, our results highlight that leaders’ capability is a crucial leader difference that determines whether AL is beneficial or detrimental to followers’ outcomes.

Beyond the theoretical implications of the present study, our findings also have several clear implications for leadership practices. First, considering the negative impact of leader authoritarianism on effectiveness evaluation and employee outcomes, leaders might reserve their authoritarian actions to counterbalance such effects, such as consulting with subordinates before making decisions, obtaining followers’ input, and explaining reasons for their decisions. In addition, organizations should pay more attention to the detrimental controlling ways of the leaders.

Second, whereas authoritarianism is unavoidable in some organizations in east Asian countries, leaders’ authoritarianism should have legitimate roots. Hence, leaders should develop themselves with professional skills and expertise when enacting centralized decision making and high control over subordinates.

Third, our findings also suggest that in addition to buffering the negative effect of authoritarian leadership, leader capability can directly enhance leaders’ effectiveness evaluations. This underlines the importance of leaders improving their personal capability to gain employees’ favorable evaluations. Moreover, it is vital for organizations to realize that there are certain conditional factors for the AL leadership styles’ efficiency, and it suggests that organizations design and implement developmental leadership programs for leaders to enhance their professional skills.

Fourth, our findings illuminate that AL decreases the leaders’ effectiveness evaluations, which in turn inhibits the followers’ task performance and affective organizational commitment. Thus, we suggest the need for practical organization interventions aimed at enhancing leaders’ effectiveness evaluations, for example, by comparing between leaders’ and followers’ perceptions of leadership effectiveness. This helps leaders to reflect on their self-awareness [81] and improves the leaders’ effectiveness.

## Figures and Tables

**Table 1:** Descriptive analysis.

Variables	Mean	SD	1	2	3	4	5	6	7	8	9	10	11
1. Age (T1)	36.58	9.89											
2. Gender (T1)	0.73	0.45	−0.004										
3. Education (T1)	13.84	2.82	−0.44 **	0.08									
4. Dyadic tenure (T1)	3.69	2.96	0.21 **	−0.18 **	0.07								
5. Power distance orientation (T1)	3.96	1.39	0.04	0.27 **	0.10	−0.01	(0.92)						
6. Leader integrity evaluation (T2)	5.70	1.18	0.09	−0.04	−0.22 **	−0.07	0.06	(0.94)					
7. Authoritarian leadership (T1)	3.07	0.77	0.05	0.12 *	0.01	0.08	0.22 **	−0.06	(0.89)				
8. Leader capability (T2)	5.82	1.12	0.06	−0.18 **	−0.19 **	−0.004	−0.12 *	0.55 **	−0.11 *	(0.91)			
9. Leader effectiveness (T2)	5.64	1.26	0.004	−0.30 **	−0.20 **	0.06	−0.34 **	0.24 **	−0.20 **	0.45 **	(0.96)		
10. Task performance supervisor (T2)	5.75	1.06	0.05	−0.33 **	−0.08	0.18 **	−0.30 **	0.16 **	−0.26 **	0.27 **	0.54 **	(0.90)	
11. Affective organizational commitment (T3)	5.04	1.25	0.09	−0.28 **	−0.21 **	0.01	−0.30 **	0.22 **	−0.13 *	0.36 **	0.64 **	0.40 **	(0.94)

Note. *N =* 341 (level 1), *N =* 99 (level 2). For gender, 0 = female, 1 = male. Coefficient alphas are in parentheses on the diagonal. * *p* < 0.05, ** *p* < 0.01.

**Table 2:** Unstandardized coefficient.

Variables	Leader Effectiveness		Task Performance		Affective Organizational Commitment	
	*Est.* (*SE*)	*Est.* (*SE*)	*Est.* (*SE*)	*Est.* (*SE*)	*Est.* (*SE*)	*Est.* (*SE*)
Intercepts	4.42 ** (0.58)	4.52 ** (0.58)	4.98 ** (0.48)	5.01 ** (0.47)	3.88 ** (0.48)	3.95 ** (0.47)
Controls						
Age	−0.01 (0.01)	−0.01 (0.01)	−0.004 (0.01)	0.00 (0.01)	0.004 (0.01)	0.01 (0.01)
Gender	−0.18 (0.14)	−0.19 (0.13)	−0.08 (0.21)	−0.01 (0.19)	−0.24 (0.21)	−0.14 (0.18)
Education	−0.02 (0.02)	−0.01 (0.02)	0.01 (0.02)	0.01(0.02)	−0.01 (0.03)	0.003(0.03)
Dyadic tenure	−0.00 (0.03)	0.01 (0.03)	0.03 (0.02)	0.03 (0.02)	−0.04 (0.03)	−0.03 (0.02)
Power distance orientation	0.01 (0.06)	0.01 (0.05)	−0.02 (0.04)	−0.02 (0.04)	−0.05 (0.06)	−0.06 (0.06)
Leader integrity evaluation	0.21 * (0.09)	0.20 * (0.09)	0.14 (0.08)	0.06 (0.06)	0.20 * (0.08)	0.08 (0.06)
Independent Variables						
Authoritarian leadership (AL)	−0.15 (0.08)	−0.15 * (0.08)	−0.27 ** (0.08)	−0.22 ** (0.08)	0.08 (0.10)	0.17 (0.09)
Leader capability (LC)	0.37 ** (0.10)	0.31 ** (0.08)	0.10 (0.08)	−0.03 (0.06)	0.22 ** (0.08)	0.00 (0.09)
Moderation Effect						
AL * LC		0.31 * (0.12)				
Mediation Effect						
Leadership effectiveness				0.36 ** (0.08)		0.58 ** (0.11)
Residual Variance	0.48 ** (0.14)	0.53 ** (0.14)	0.44 ** (0.10)	0.45 ** (0.10)	0.28 * (0.13)	0.33 ** (0.13)
Pseudo R^2^	0.24	0.27	0.14	0.21	0.14	0.30

Note. *N* = 341 (level 1), *N* = 99 (level 2). * *p* < 0.05, ** *p* < 0.01.

## Data Availability

The data presented in this study are available on request from the corresponding author.
